# The Evaluation of Chinese Therapeutic Food for the Treatment of Moderate Dyslipidemia

**DOI:** 10.1155/2012/508683

**Published:** 2012-03-14

**Authors:** Shu Sun, Hong Xu, Lawrence Ngeh

**Affiliations:** ^1^School of Biomedical and Health Sciences, Victoria University, P.O. Box. 14428, Melbourne, VIC 8001, Australia; ^2^School of Engineering and Science, Victoria University, P.O. Box 14428, Melbourne, VIC 8001, Australia

## Abstract

The clinical efficacy of the Chinese therapeutic food (specifically hawthorn fruit and Chinese kiwifruit-extract compound) on dyslipidemia was evaluated in this placebo-controlled, double blind, paired clinical trial conducted in Melbourne, Australia. Forty-three participants diagnosed with moderate dyslipidemia and met the study criteria were randomly assigned to Group A or B, with baseline characteristics matched. Twenty-seven participants completed all the tests, the blood lipid profile including total cholesterol (TC), high-density lipoprotein cholesterol (HDL-c), low-density lipoprotein cholesterol (LDL-c), and triglycerides (TG) was analysed. The traditional Chinese medicine diagnosis was made based on participants' symptoms and signs. The results indicate that a four-week intake of the compound increased the serum HDL-c levels by 5% (*P* = 0.026) and decreased the ratios of TC/HDL-c and LDL-c/HDL-c (*P* = 0.012 and *P* = 0.044, resp.). The placebo intake did not significantly change the blood lipid profile. In the initial 43 participants with dyslipidemia, 76.7% of them were diagnosed with “*Spleen* deficiency” and 58.1% with “*Liver qi* stagnation.” The intake of hawthorn fruit and Chinese kiwifruit extract compound may increase the serum levels of HDL-c and decrease the ratios of TC/HDL-c and LDL-c/HDL-c, therefore, may reduce the risk of cardiovascular disease.

## 1. Introduction

Atherosclerosis and subsequent cardiovascular disease (CVD) are common and have high mortality. Dyslipidemia is considered to be responsible for the development of atherosclerosis through blood lipid accumulation and oxidation [1, 2]. The atherogenic lipid profile includes increased low-density lipoprotein cholesterol (LDL-c), triglycerides (TG), and decreased high-density lipoprotein cholesterol (HDL-c), which are all recognized as independent risk factors for CVD. Therapeutic lifestyle changes are recommended as the first choice for blood lipid management, and dietary intake is suggested to include reduced intake of saturated fat and increased LDL-c lowering nutrients [3]. HMG-CoA reductase inhibitors (statins), bile acid sequestrants, nicotinic acid, and fibric acids, are conventionally employed to achieve different goals of blood lipid management. However, side effects of the agents, such as myopathy and increased liver enzymes [4], are a concern to users and clinicians.

Chinese therapeutic food may make a valuable contribution to a more balanced diet. They have been used widely with acknowledged safety in Chinese history, and recipes based on herbal dietary therapy are accepted extensively nowadays for the prevention and treatment of heart diseases [5]. Chinese people have traditionally preferred food and herbs to drugs for health care, and many herbs are described as both food and medicine by the Chinese Ministry of Health [6]. In the selected therapeutic food supplement, hawthorn fruit (*Shan Zha*) is known in traditional Chinese medicine (TCM) for its effects on reducing food stagnancy and* blood *stasis. As herbal medicine, it is also used to treat dyslipidemia, angina pectoris, and hypertension [7]. Chinese kiwifruit (*Zhong Hua Mi Hou Tao*) originated from China and its various cultivars have been widely consumed globally. Previous studies have shown that the Chinese kiwifruit cultivar used in the supplement for this study appears to be antiatherogenic [8, 9].

This study evaluated the efficacy of the selected Chinese therapeutic food supplement for the treatment of dyslipidemia in Australia and compared the outcomes with the previous study conducted in China [10].

## 2. Methods

### 2.1. General Description

A placebo-controlled, double-blind, paired research design was used in the present study, based upon a previous study with positive outcomes [10]. Among the 62 applicants, 43 Australian participants who met the inclusive criteria were recruited and assigned into two groups. Two interventions, the therapeutic food compound and the placebo, were applied in this study. The intervention in the two groups was swapped at the middle of the study. Before and after each period of intervention, fasting blood samples were taken and analysed for the serum lipid levels. Participants were also assessed from the TCM perspective.

This study was approved by the Human Research Ethics Committee of Victoria University, Melbourne, Australia. It was registered with the Australian-New Zealand Clinical Trials Registry. The Therapeutic Food Administration (TGA) was notified of this trial.

In this present study, the heavy metal contents in the compound were analysed by the Australian National Measurement Institute and were found to be safe for human consumption. The pesticide concentrations of the supplements used in this study were also analysed at Victoria University and found to be safe for human consumption.

### 2.2. Participants

All participants met the following criteria before enrollment:

- have lived in Australia for the past 10 years,
- diagnosed with moderate dyslipidemia, in which fasting serum LDL-c level within 3.2–4.6 mmol/L (125–180 mg/dL) or TG 2.0–2.9 mmol/L (180–250 mg/dL), according to Adult Treatment Panel III [3],
- aged 40–70 years and with blood pressure lower than 159/99 mmHg (A meta-analysis indicated that older age and increasing blood pressure could attenuate the proportional CVD risk reduction of cholesterol lowering [11]; a similar clinical trial to evaluate the effects of berry consumption on CVD risk factors, using “140–159 mmHg systolic blood pressure or 90–99 mmHg diastolic blood pressure” as inclusive criteria for blood pressure, has shown favourable outcomes [12]),
- absence of other major medical conditions (i.e., established CVD, severe diabetes, thyroid dysfunction, asthma, hepatic or renal disorders) or pregnancy,
- not have taken other supplements or medicine which may influence blood lipid levels in the past 3 months,
- not allergic to the extract of the therapeutic food or wheat germ,
- not have fluid retention or severe diarrhoea (according to the TCM theory, the nature of kiwifruit is considered as *cool*, which means that intake of kiwifruit may give rise to or deteriorate fluid retention for people with very weak digestive system and cold nature; hawthorn fruit may cause slight diarrhoea due to its effect on reducing food stagnancy, however, it is unlikely occur in this study because kiwifruit's cool nature can be balanced by hawthorn fruit's warm nature and the nourishing effect of kiwifruit may also balance the hawthorn fruit's effect of reducing stagnancy).

This study purposely did not include participants with an Eastern Asian cultural background as a similar study has already been conducted in China [10]. It is considered the people with an Eastern Asian background, such as Chinese, Korean, and Japanese, who may share similar culture-affected life styles. Therefore, genetic factors, locality, and/or cultural differences were taken into account in comparison with the previous study.

Volunteers participating in this study were recruited from the staff of Victoria University and residents from the local community (Melbourne, Australia). They became aware of this study through public e-mails within Victoria University, posters at community health centers, local newspapers, and word of mouth.

The issues pertaining to this research were reinforced and explained by the researchers at each interview. The treatment and control crossover procedure was clearly described and explained to each participant. The participants were also informed that they could withdraw from this study at any time. Each participant signed the consent form prior to recruitment. All records were kept secure and confidential and can only be accessed by the related researchers.

### 2.3. Study Design

Participants were assigned into pairs according to the baseline blood lipid levels and demographic characteristics (i.e., age and gender). A random number table was used for grouping the randomly allocated participants. Participants in Group A received the treatment of the supplement for the first four weeks while participants in Group B received the control supplement (placebo) during the same period. For the second four weeks, the interventions were switched, that is, Group B received the supplement and Group A took placebo, to observe long-term treatment effects in Group A. The total duration of participation was eight weeks. Three office visits were organised during the study (at the beginning, mid and the end of the participation, resp.). Treatment effects have been considered within individual Group A and B, and also for the combination of Group A + B.

A practitioner who was familiar with this type of clinical trial was invited as the third party to label the treatment and placebo powder (which were packed in the same container). The powder was assigned to match the participants' codes and groups (i.e., A1, A2…, or B1, B2…; names were kept confidential). This practitioner was asked not to reveal relevant information to either researchers or participants until the final data analysis was completed.

Blood lipid levels can be affected by lifestyle-related factors and can fluctuate over time without treatment. All participants in both the treatment and control groups were advised not to change their normal daily lifestyle during the course of this study. Thus, they were asked to complete a questionnaire as to record their diets and physical activities for the first and last three days in each phase of their participation. The questionnaire allowed the researchers to monitor the participants' diet and physical activity to determine whether there were significant changes. By completing the questionnaire, the participants were also made aware of their habits and were reminded not to make any changes during the study.

### 2.4. Intervention

Hawthorn fruit compound (HFC) with an established safe and effective dosage of 10 g twice per day [7, 10] was selected as the treatment supplement. The compound includes Chinese therapeutic food extract hawthorn fruit (*Crataegus pinnatifida Bge*.) and Chinese kiwifruit (*Actinidia chinensis var. deliciosa*). It contains the effective components haw flavone, triterpenoid, rutin, tartaric acid, citric acid, crategeolic acid, ester, glucoside, analytical lipid enzyme, carbohydrate, some saponins, polysaccharides, multiple kinds of organic acids, isoflavones, and adequate amounts of trace elements, for example, zinc (Zn) and strontium (Sr). The placebo consists of wheat germ, food dye, and citric acid, and sweetener was used as a control supplement in the same dose. Both the treatment and placebo drinking powder were provided by the supplier of the previous study in China [7, 10]. Participants were asked to mix the drinking powder (either treatment or placebo) with warm water, and to take the supplement with an interval of minimum one hour after food intake. Each participant was asked to complete a checklist of everyday supplement intake to monitor the progress.

### 2.5. Blood Lipid Test Parameters

Blood lipid levels including TC, TG, and HDL-c were assayed during pre-, mid- (at the end of 4th week), and posttreatment periods. The LDL-c level was calculated using the Friedewald equation [13]: LDL-c = TC-HDL-c−TG/2.2 (all values in mmol/L). The ratios of TC/HDL-c and LDL-c/HDL-c were also investigated.

Blood samples were taken in the Outpatient Department of the appointed hospitals with Melbourne Health Shared Pathology Service (MHSPS), including Royal Melbourne Hospital, Footscray Western Hospital, and Sunshine Hospital. All blood tests were performed in the Melbourne Health Shared Pathology Laboratory by the appointed staff. Enzymatic and spectrophotometric methods were used for the study assays. Participants were asked to fast 12 hours before the blood sample collection.

### 2.6. Traditional Chinese Medicine Assessment

Participants' symptom information was collected using the standard TCM diagnostic methods, including inspection, asking questions, listening, smelling, and palpation. The occurrence of certain symptoms and signs indicates certain patterns of disharmony, which was determined at every visit of assessment.

### 2.7. Statistical Analysis

The data were analysed through the application of paired *t-*test using a software program SPSS 18.0 to determine the differences between pre- and postinterventions, and *P* < 0.05 was accepted as being significant.

## 3. Results

### 3.1. General Information

**Figure 1:**
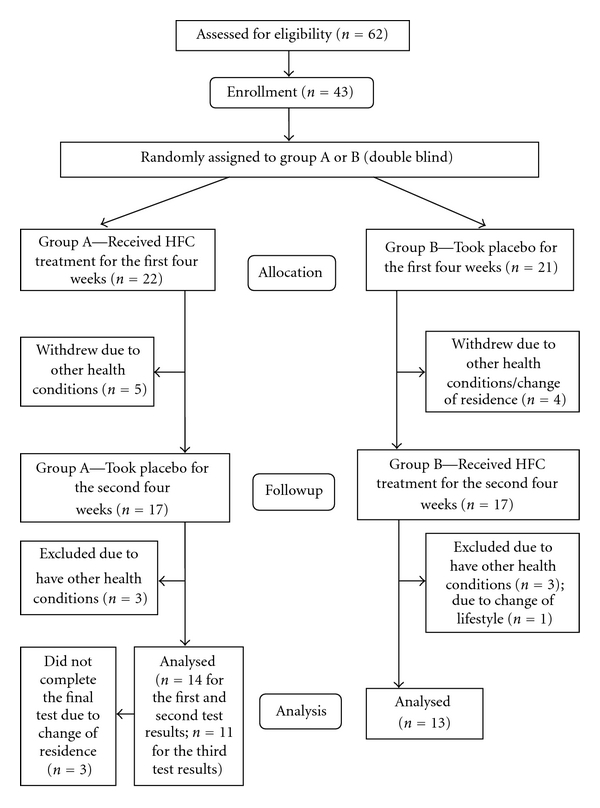
Flow chart of participants through each stage of the trial.

Twenty-seven of the 43 participants completed all the treatment and placebo intervention applicable to this study in eight weeks (Table 1). There is no significant difference of age, gender, baseline serum LDL-c levels, and blood pressure between the two groups at the baseline level. In Group A, 11 participants completed all the three blood tests; three participants did not complete the third blood test. In Group B, 13 participants completed all the tests. The participants who discontinued were affected by conditions not related to this study, such as moving to places other than Melbourne, other health conditions, and significant lifestyle changes (Figure 1).

### 3.2. Serum High-Density Lipoprotein Cholesterol (HDL-c) Levels

There is an increase of the HDL-c levels when comparing the pre- and posttherapeutic food treatment in Group A. However, the change is not statistically significant (*P* = 0.09). When combining the treatment results from both groups (Group A + B), it was found that the overall increase of HDL-c is statistically significant (*P* = 0.026, Table 2). This indicates that the HFC intake can improve the HDL-c level. If a larger sample size is used, the result is likely to be more promising.

There was no significant difference of the HDL-c levels between pre- and postplacebo intake in either Group A or Group B (Table 3), although a decreasing trend is observed. This indicates that the placebo intake may not affect the HDL-c level significantly in this study. It is noted that in the participants of Group A, who had completed their HFC treatment prior to placebo intake, the HDL-c levels appear to decrease not as much as compared to the results of the participants of Group B, who meanwhile took placebo first. Further research with more participants is required to evaluate the long-term effect of HFC on the HDL-c levels.

### 3.3. Serum Total Cholesterol (TC) and Triglycerides (TG)

Tables 4 and 5 show the effects of either HFC or the placebo on TC and TG are not statistically significant (*P* > 0.05).

### 3.4. The Ratio of Total Cholesterol and High-Density Lipoprotein Cholesterol (TC/HDL-c)

Table 6 shows that there is a significant decrease of the TC/HDL-c ratio comparing the pre- and posttreatment in Group B (*P* = 0.032) and in Group A + B (*P* = 0.012). The results indicate that HFC can effectively lower the ratio of TC/HDL-c, an informative marker of atherosclerosis.

There is an insignificant increase of the TC/HDL-c ratio, when comparing preplacebo (post-HFC treatment) and postplacebo in Group A. However, an increase of the TC/HDL-c ratio is statistically significant (*P* = 0.047) in Group B without the impact of HFC treatment (Table 7). This may indicate a “natural” increase of TC in Group B if no treatment is provided.

### 3.5. Serum Low-Density Lipoprotein Cholesterol (LDL-c)

There is an insignificant difference of LDL-c levels comparing pre- and post-HFC treatment in Group A, Group B and overall Group A + B (*P* > 0.05) (Table 8).

Again there is an insignificant increase of the LDL-c level comparing the pre- and postplacebo intake in both Group A and Group B (*P* > 0.05), which indicates that the placebo intake did not have a significant impact on the LDL-c level in this study (Table 9).

### 3.6. Ratio of LDL-c/HDL-c

Similar to the ratio of TC/HDL-c, the ratio of LDL-c/HDL-c provides a more informative marker for CVD risk than the individual value of LDL-c and HDL-c. There is a decreasing trend of the LDL-c/HDL-c ratio after the treatment in Group A and Group B (Table 10). When combining the results from both groups, the decrease in the LDL-c/HDL-c ratio is statistically significant (*P* = 0.044).

There is an insignificant increase of LDL-c/HDL-c ratio comparing posttreatment and postplacebo in Group A. However, in Group B, without the impact of HFC treatment, the ratio increased significantly after the placebo intake (*P* = 0.04, Table 11). This may indicate a “natural” increase of LDL-c/HDL-c ratio in Group B if no treatment is provided.

### 3.7. General Changes of the Blood Lipid Profile

The results of the study show possible benefits of HFC on regulating blood lipid levels.  Table 12 summarizes the changes of the blood lipid levels when comparing pre- and posttreatments in the two groups.

### 3.8. Baseline Characteristics of TCM Assessment-Identification of TCM Patterns

The following subjective symptoms were described by the participants: lower back pain, knee/leg/foot pain, poor appetite, abdominal discomfort, diarrhoea, constipation, tiredness, palpitations, insomnia, irritability/anxiety/stress, headache, and thirst. The main manifestations of the common patterns which may be seen in patients with dyslipidemia are summarized in Table 13. The distributions of patterns, major symptoms, signs of tongue and pulse are summarized in Tables 14, 15, 16, and 17. The most prevalent symptoms of the 43 participants are “abdominal symptoms” (53.5%) “thirst” (51.2%) and “abnormal bowel movement” (46.5%).

Among the 43 participants who were initially recruited, the TCM *Spleen *deficiency was found to be predominant (76.7% of the total). Symptoms and signs which may be relevant to *Spleen *deficiency are prevalent in all the participants. For example, more than half of the participants complained of abdominal symptoms before participation; 37.3% of them had loose stool, diarrhea, or occasional constipation and diarrhea; 39.5% of them prefer a warm condition to a cool one. In addition, 48.8% of the participants had teeth marks on their tongues and more than half had weak pulse. These signs on the tongue and the pulse are usually indicative of *Spleen *deficiency. It was found that 58.1% of the participants were diagnosed of *Liver qi *stagnation. 55.8% of the participants complained of stress and 51.2% of them had wiry pulse, which supports the *Liver qi *stagnation diagnosis.

## 4. Discussion

### 4.1. The Effects on HDL-c

In the present study, the four-week intake of HFC appears to effectively increase the serum levels of HDL-c by 5% (Group A + B) for the 27 participants. Compared with the previous clinical study on this compound [10], which has achieved the +7% increase of HDL-c, the increase of 5% in the present study is reasonable. The HDL-c changes are not statistically significant when comparing pre- and postplacebo intakes in both groups, a decreasing trend (5%) has been observed. Because of limited sample size, further research with more participants is required.

Epidemiologic studies have demonstrated a high cardiovascular risk at low levels of HDL-c regardless of the LDL-c levels [14]. Substantial atherosclerotic regression (*⩾*5% decrease in atheroma volume) occurred only in patients achieving both low levels of LDL-c (<2.26 mmol/L) and an increase of 7.5% in HDL-c with lipid lowering therapy [15].

According to Ma et al. [16], the component of triterpenoid acid from hawthorn fruit can enhance the activity of HDL receptor, which may be a possible mechanism for the effect on HDL-c. Kiwifruit intake could also contribute to an increase of HDL-c level in the present study. In a previous clinical trial, forty-three participants with dyslipidemia consumed two kiwifruit per day for eight weeks, when comparing both pre- and postintervention, the HDL-c concentration has significantly increased [17].

### 4.2. The Effects on LDL-c, and Ratios of TC/HDL-c and LDL-c/HDL-c

In this present study, HFC induced a significant decrease of the TC/HDL-c ratio and LDL-c/HDL-c ratio. However, no significant effects on serum TC, LDL-c, and TG were found. Such outcomes are consistent with the previous study [17].

Although the LDL-c is considered as the primary treatment target for lipid management [3, 18], the CVD-predicting power of TC/HDL-c ratio and LDL-c/HDL-c ratio has been investigated extensively. A meta-analysis of 61 observational studies has shown that the TC/HDL-c ratio is substantially more informative as a predictor of CHD mortality than the individual value of TC, HDL-c, or non-HDL-c [11]. In a post hoc study with 9770 participants, it was indicated that the LDL-c/HDL-c ratio was highly predictive of major cardiovascular events [14].

Another relevant study showed that HFC significantly reduced serum levels of LDL-c, TG, and the LDL-c/HDL-c ratio in atherosclerotic mice, and this effect was comparable with the control of statins therapy [19].

In the previous clinical study conducted by Chen et al. [10], the HFC drink was taken by 60 Chinese participants for 31 days. The results showed that the supplement significantly improved the whole blood lipid profile, including reducing TC (−10%), TG (−12%), LDL-c (−18%), and increasing HDL-c (+7%). Statistically, the design and results of the previous study was used to predict the statistical power of the present study. The power calculation indicated that 62 participants were needed to achieve overall statistically significant outcomes. The insignificant results of LDL-c and TG levels in this study may be due to the low number of participants.

### 4.3. Impacts of Genetic, Ethnical, Cultural, and Lifestyle-Related Factors

In the present study, the Australian participants originated from more than 10 countries or areas (not including East Asia), which reflects the fact of the composition of Melbourne population, however, this may also affect the research outcomes because of the diversity of genetic factors and culture-related lifestyles. Classification of participants by different lipoprotein subgroups or relevant genotypes was unavailable. It is difficult to determine whether there was a detectable diet-gene interaction involved in the study or not. In the case of disparities of blood lipid level and CVD risk, evidence on their relationships with ethnic features is not convincing.

In Chen et al. study [10], the HFC drink significantly increased the serum levels of apoA—I and superoxide dismutase (SOD), and decreased the levels of apoB and malondialdehyde (MDA) in the Chinese participants. Such results show the additional favorable effects of HFC on cardiovascular health. The serum apoB level represents the total atherogenic property of blood lipids, and the apoA—I level reflects the antiatherogenic property. The MDA and SOD levels indicate the body's oxidative status and antioxidative ability, respectively. Further study with other biomedical parameters is of benefit to investigate the effects of HFC.

### 4.4. Baseline Characteristics of TCM Assessment

The above TCM findings are consistent with previous research in which *Spleen *deficiency and *Liver qi *stagnation were recognized as major root causes of abnormal blood lipid metabolism [20, 21]. This may indicate that high-level stress and reduced digestion associate with the occurrence of dyslipidemia. The same pattern was found in Australian women with premenstrual syndromes [22]. However, because the data of TCM pattern prevalence in general Australian population are unavailable, it is difficult to determine whether the pattern features observed in this study is specific for the whole cohort with moderate dyslipidemia or not. Studies on TCM pattern prevalence in Australian population are needed and the results can be used as a reference for outcome evaluation of the TCM clinical trials.

Long-term or severe dyslipidemia have also been viewed as a condition or pattern of *blood *and *phlegm *stagnation in TCM. It is noted that 32.6% of the participants have the purple tongue body, a typical sign indicating *blood stasis*. However, the prevalence of *phlegm *and *blood* stagnation pattern is not as high compared to *Spleen* deficiency in the participants with moderate dyslipidemia. Therefore, the pattern of *blood *and *phlegm* stagnation may need to be identified in severe dyslipidemia cases.

Another finding of this study is that many participants had *heat*-related symptoms and signs, that is, 51.2% of the participants complained of thirst or dry mouth, 34.9% with red tongue tip or yellow tongue coating, and 44.2% with tongue coating fissure. This *heat* pattern was also reported by Fu and Xu [23] in their study on Australian peri-menopausal women. Although *Spleen *deficiency may also result in thirst as the fluid cannot be transformed sufficiently and be transported upward to nourish the mouth, about one third of the participants were diagnosed of *Heart heat* pattern or *Stomach yin* deficiency pattern, respectively. *Heat* patterns in patients with dyslipidemia have not been emphasized in previous TCM research. Possible TCM mechanisms may involve the *qi* stagnation and *phlegm *accumulation which can generate* heat, *resembling the *heat*-generating process of compost; the *heat* may in turn deteriorate lipid metabolism by overheating *fluid *into the thick and pathological *phlegm.* According to the TCM theory, kiwifruit, with the cool nature, can remove *heat *and nourish* yin *[24]. Thus, the consumption of kiwifruit can be beneficial for people with *heat* patterns. Further study is needed to explore the optimized the TCM treatment for people with dyslipidemia and *heat *pattern. The utilization of Chinese therapeutic food according to the TCM differential diagnosis can be explored as a tailored treatment of diet-related conditions.

## 5. Conclusion

Intake of the Chinese therapeutic food, specifically the hawthorn fruit compound, has shown a positive effect on increasing serum HDL-c levels and decreasing ratios of TC/HDL-c and LDL-c/HDL-c, which indicates an improvement of the blood lipid profile. As a dietary therapy, this compound can be considered for the treatment of hyperlipidemia and the prevention of atherosclerosis, and such effects need to be further explored. The major TCM patterns observed in this moderately dyslipidemic participants' group include “*Spleen* deficiency” and “*Liver qi *stagnation.”

## Figures and Tables

**Table 1:** Participants' general information.

^#^Group (No)	Age* Mean ± SD	Male	Female	LDL-c* Mean ± SD	Systolic blood pressure*(mmHg) Mean ± SD	Diastolic blood pressure* (mmHg) Mean ± SD
A (*n* = 14)	56.00 ± 7.09	5	9	4.01 ± 0.60	125.1 ± 13.4	77.9 ± 7.5
B (*n* = 13)	53.62 ± 9.85	8	5	3.95 ± 0.61	125.8 ± 10.0	79.9 ± 7.3

^#^A: Group A—took HFC (treatment) for the first four weeks; then took the placebo for the second four weeks; B: Group B—took the placebo for the first four weeks; then took the HFC for the second four weeks.

*P > 0.05.

**Table 2:** Comparison of HDL-c levels (mmol/L) between pre- and post-HFC treatment.

Group (no)	Pretreatment* Mean ± SD	Posttreatment** Mean ± SD
A (*n* = 14)	1.44 ± 0.33	1.53 ± 0.42^#^
B (*n* = 13)	1.50 ± 0.42	1.54 ± 0.48
A + B (*n* = 27)	1.47 ± 0.37	1.54 ± 0.44^##^

^#^ *P* = 0.09; ^##^ *P* = 0.026.

*Pretreatment: Group A—pre-HFC treatment; Group B—completed the first four weeks of the placebo intake and is to start HFC treatment, that is Postplacebo value.

**Posttreatment: Group A completed HFC treatment for the first four weeks, that is Preplacebo value; Group B completed the HFC treatment for the second four weeks.

**Table 3:** Comparison of HDL-c levels (mmol/L) between pre- and postplacebo intake.

Group (no)	Preplacebo intake* Mean ± SD	Postplacebo intake** Mean ± SD
A (*n* = 11)	1.55 ± 0.39***	1.53 ± 0.34
B (*n* = 13)	1.58 ± 0.44	1.50 ± 0.42

*P* > 0.05.

*Preplacebo intake: Group A completed HFC treatment for the first four weeks and started the placebo intake.

**Postplacebo intake: Group A completed the placebo intake for the second four weeks; Group B completed the placebo intake for the first four weeks.

***This value is different from the value of Group A (*n* = 14) at posttreatment (1.53 ± 0.42, Table 2) because three participants withdrew from the trial. Eleven participants in Group A (*n* = 11) completed their test.

**Table 4:** Comparison of TC and TG levels (mmol/L) between pre- and post-HFC treatment.

Group (No)	TC Pretreatment* Mean ± SD	TC Posttreatment** Mean ± SD	TG Pretreatment* Mean ± SD	TG Posttreatment** Mean ± SD
A (*n* = 14)	6.23 ± 0.63	6.34 ± 0.57	1.69 ± 0.79	1.76 ± 0.89
B (*n* = 13)	6.41 ± 0.60	6.28 ± 0.70	1.74 ± 0.83	1.55 ± 0.71
A + B (*n* = 27)	6.31 ± 0.61	6.31 ± 0.62	1.71 ± 0.79	1.66 ± 0.80

*P* > 0.05.

*Pretreatment: Group A—pre-HFC treatment; Group B—completed the first four weeks of the placebo intake.

**Posttreatment: Group A completed HFC treatment for the first four weeks; Group B completed HFC treatment for the second four weeks.

**Table 5:** Comparison of TC and TG levels (mmol/L) between pre- and postplacebo intake.

Group (No)	TC Preplacebo intake* Mean ± SD	TC Postplacebo intake** Mean ± SD	TG Preplacebo intake* Mean ± SD	TG Postplacebo intake** Mean ± SD
A (*n* = 11)	6.40 ± 0.60	6.57 ± 0.65	1.54 ± 0.59	1.59 ± 0.73
B (*n* = 13)	6.35 ± 0.65	6.41 ± 0.60	1.83 ± 1.00	1.74 ± 0.83

*P* > 0.05.

*Preplacebo intake: Group A completed HFC treatment for the first four weeks and started the placebo intake.

**Postplacebo intake: Group A completed the placebo intake for the second four weeks; Group B completed the placebo intake for the first four weeks.

**Table 6:** Comparison of the TC/HDL-c ratio between pre- and post-HFC treatment.

Group (No)	Pretreatment*	Posttreatment**
	Mean ± SD	Mean ± SD
A (*n* = 14)	4.54 ± 1.12	4.41 ± 1.13
B (*n* = 13)	4.58 ± 1.26	4.37 ± 1.20^#^
A + B (*n* = 27)	4.56 ± 1.17	4.39 ± 1.14^##^

^#^ *P* = 0.032; ^##^ *P* = 0.012.

*Pretreatment: Group A—pre-HFC treatment; Group B—completed the first four weeks of the placebo intake.

**Posttreatment: Group A completed HFC treatment for the first four weeks; Group B completed HFC treatment for the second four weeks.

**Table 7:** Comparison of the TC/HDL-c ratio between pre- and postplacebo intake.

Group (No)	Preplacebo intake*	Postplacebo intake**
	Mean ± SD	Mean ± SD
A (*n* = 11)	4.29 ± 0.80	4.44 ± 0.93^#^
B (*n* = 13)	4.31 ± 1.20	4.58 ± 1.26^##^

^#^ *P* > 0.05; ^##^ *P* = 0.047.

*Preplacebo intake: Group A completed HFC treatment for the first four weeks and started the placebo intake.

**Postplacebo intake: Group A completed the placebo intake for the second four weeks; Group B completed the placebo intake for the first four weeks.

**Table 8:** Comparison of LDL-c levels (mmol/L) between pre- and post-HFC treatment.

Group (No)	Pretreatment*	Posttreatment**
	Mean ± SD	Mean ± SD
A (*n* = 14)	4.01 ± 0.60	4.03 ± 0.43
B (*n* = 13)	4.10 ± 0.50	4.02 ± 0.63
A + B (*n* = 27)	4.06 ± 0.55	4.02 ± 0.53

*P* > 0.05.

*Pretreatment: Group A—pre-HFC treatment; Group B—completed the first four weeks of the placebo intake.

**Posttreatment: Group A completed HFC treatment for the first four weeks; Group B completed HFC treatment for the second four weeks.

**Table 9:** Comparison of LDL-c levels (mmol/L) between pre- and postplacebo intake.

Group (No)	Preplacebo intake*	Postplacebo intake**
	Mean ± SD	Mean ± SD
A (*n* = 11)	4.17 ± 0.33	4.32 ± 0.50
B (*n* = 13)	3.95 ± 0.61	4.10 ± 0.50

*P* > 0.05.

*Preplacebo intake: Group A completed HFC treatment for the first four weeks and started the placebo intake.

**Postplacebo intake: Group A completed the placebo intake for the second four weeks; Group B completed the placebo intake for the first four weeks.

**Table 10:** Comparison of the LDL-c/HDL-c ratio between pre- and post-HFC treatment.

Group (No)	Pretreatment*	Posttreatment**
	Mean ± SD	Mean ± SD
A (*n* = 14)	2.94 ± 0.82	2.81 ± 0.71
B (*n* = 13)	2.94 ± 0.87	2.83 ± 0.94^#^
A + B (*n* = 27)	2.94 ± 0.83	2.81 ± 0.82^##^

^#^ *P* = 0.075; ^##^ *P* = 0.044.

*Pretreatment: Group A—pre-HFC treatment; Group B—completed the first four weeks of the placebo intake.

**Posttreatment: Group A completed HFC treatment for the first four weeks; Group B completed HFC treatment for the second four weeks.

**Table 11:** Comparison of the LDL-c/HDL-c ratio between pre- and postplacebo intake.

Group (No)	Preplacebo intake*	Postplacebo intake**
	Mean ± SD	Mean ± SD
A (*n* = 11)	2.80 ± 0.53	2.91 ± 0.60^#^
B (*n* = 13)	2.68 ± 0.80	2.94 ± 0.87^##^

^#^ *P* > 0.05; ^##^ *P* = 0.041.

*Preplacebo intake: Group A completed HFC treatment for the first four weeks and started the placebo intake.

**Postplacebo intake: Group A completed the placebo intake for the second four weeks; Group B completed the placebo intake for the first four weeks.

**Table 12:** Changes of the blood lipid profile comparing pre- and post-HFC treatment.

Blood Lipid	Group A	Group B	Group A + B
Total cholesterol (TC)	→	→	→
Low-density lipoprotein cholesterol (LDL-c)	→	→	→
High-density lipoprotein cholesterol (HDL-c)	↑	→	↑
The ratio of TC/HDL-c	→	↓	↓
The ratio of LDL-c/HDL-c	→	→	↓
Triglycerides (TG)	→	→	→

Note: ↑ = significant increase.

→ = no significant change.

↓ = significant decrease.

**Table 13:** Major TCM patterns of disharmony and clinical manifestations relating to dyslipidemia.

Patterns	Main clinical manifestations
*Spleen* deficiency	Tiredness, preference of warmth, abdominal distension/discomfort, reduced appetite, indigestion, loose stool/diarrhea (or alternately with constipation), pale or swollen tongue with or without teeth marks, weak (and/or slippery) pulse

*Liver qi *stagnation	Stress, depression, irritability, anxiety, migraine/dizziness, abdominal distension, oppression in the chest, irregularity of menstruation, wiry/rough pulse

*Kidney* deficiency	Tiredness, headache/dizziness, insomnia, lower back pain, knee/leg pain, weak pulse in the cubit *(chi)*; *yin* deficiency with feeling of heat/irritability, thirst, night sweating, thin and red tongue with dry/little coating, thin and/or rapid pulse; *qi* and *yang *deficiency with preference of warmth, pale tongue with white coating, weak pulse (especially in the cubit)

*Phlegm* and* damp* accumulation	Obesity, dizziness, feeling of heavy head and/or body, fullness in the chest and/or abdomen, nausea, bland taste in the mouth, thirst but without intention to have water, greasy tongue coating, slippery pulse

*Blood *stasis	Dark complexion/lips, encrusted skin, fixed stabbing pain, clots in the menstrual blood with/without irregularity of menstruation, purple tongue, rough/intermittent/bound pulse

**Table 14:** Frequencies of baseline TCM patterns of the 43 participants.

TCM patterns*	Frequency	Proportion of the total (%)
*Spleen* deficiency	33	76.7
*Liver qi* stagnation	25	58.1
*Kidney yin* deficiency	9	20.9
*Kidney qi *deficiency	4	9.3
*Phlegm *and* damp* accumulation	16	37.2
*Blood *stasis	13	30.2
Food stagnation	6	14.0
*Heart heat*	14	32.6
*Heart qi* deficiency	5	11.6
*Stomach yin* deficiency	15	34.9

*Manifestations of different TCM patterns may coexist in one individual.

**Table 15:** Frequencies of major baseline symptoms of the 43 participants.

Major symptoms	Frequency	Proportion of the total (%)
Preference of warmth	17	39.5
Preference of coolness	9	20.9
Night sweating	5	11.6
Thirst/Dry mouth	22	51.2
Poor appetite	5	11.6
Abdominal symptoms	23	53.5
Abdominal distension	14	32.6
Other discomfort	13	30.2
Reflux	11	25.6
Abnormal bowel movement	20	46.5
Loose stool/diarrhea	10	23.3
Constipation	4	9.3
Alternate diarrhea and constipation	6	14.0
Feeling of oppression/occasional pain in the chest	6	14.0
Palpitation	7	16.3
Feeling stressful/anxiety/depression	24	55.8
Sleep disorder	23	53.5
Feeling sleepy	13	30.2
Dizziness/feeling light-headed	2	4.7
Headache	11	25.6
Lower back/leg/knee pain	16	37.2

**Table 16:** Tongue features of the 43 participants.

Tongue feature	Frequency	Proportion of the total (%)
*Tongue body color*		
Pink	6	14.0
Pale	12	27.9
Red	6	14.0
Purple	14	32.6
Red tip	15	34.9

*Tongue body *		
Normal	13	34.9
Swollen	6	14.0
Thin	22	51.2
With teeth marks	21	48.8
Without teeth marks	22	51.2

*Coating texture*		
Thin	7	16.3
Thick/greasy	9	20.9
Dry	6	14.0
Less than normal	3	7.0
Fissure	19	44.2

*Coating color*		
White	28	65.1
Yellow	15	34.9

**Table 17:** Pulse features of the 43 participants.

Pulse feature	Frequency	Proportion of the total (%)
*Rate*		
Normal	33	76.7
Slow	1	2.3
Fast	6	14.0
Uneven	2	4.6

*Left pulse*		
Slippery	15	34.9
Thin	15	34.9
Soft/weak	23	53.5
Wiry	22	51.2

*Right pulse*		
Slippery	18	41.9
Thin	19	44.2
Soft/Weak	25	58.1
Wiry	15	34.9
